# Systemic Inflammation during and after Bronchiectasis Exacerbations: Impact of *Pseudomonas aeruginosa*

**DOI:** 10.3390/jcm9082631

**Published:** 2020-08-13

**Authors:** Rosario Menéndez, Raúl Méndez, Isabel Amara-Elori, Soledad Reyes, Beatriz Montull, Laura Feced, Ricardo Alonso, Rosanel Amaro, Victoria Alcaraz, Laia Fernandez-Barat, Antoni Torres

**Affiliations:** 1 Pulmonary Medicine Department, Hospital Universitario y Politécnico La Fe, 46023 Valencia, Spain; rmendezalcoy@gmail.com (R.M.); milisaelori@hotmail.com (I.A.-E.); reyes@comv.es (S.R.); gelina82@hotmail.com (B.M.); l.fecedolmos@gmail.com (L.F.); 2 CIBER Enfermedades Respiratorias (CIBERES), 28029 Madrid, Spain; RAMARO@clinic.cat (R.A.); victoriaalcarazserrano@gmail.com (V.A.); lfernan1@clinic.cat (L.F.-B.); ATORRES@clinic.cat (A.T.); 3 Laboratory Department, Hospital Universitario y Politécnico La Fe, 46023 Valencia, Spain; alonso_ricdia@gva.es; 4 Pulmonary Medicine Department, Hospital Clínico y Provincial, IDIBAPS, 08036 Barcelona, Spain

**Keywords:** bronchiectasis, inflammation, exacerbation

## Abstract

Bronchiectasis is a chronic structural disease associated with exacerbations that provoke systemic inflammation. We aimed to evaluate the systemic acute proinflammatory cytokine and its biomarker profiles during and after exacerbations and its relationship with the severity of episode, microbiological findings, and the bronchiectasis severity index. This prospective observational study compared exacerbation and stable groups. Cytokine (interleukins (IL)-17a, IL-1β, IL-6, IL 8; tumor necrosis factor-alpha (α)) and high-sensitivity C-reactive protein (hsCRP) levels were determined by multiplex analysis on days 1, 5, 30, and 60 in the exacerbation group and on day 1 in the stable group. We recruited 165 patients with exacerbations, of which 93 were severe (hospitalized). Proinflammatory systemic IL-17a, IL-1β, IL-8, and tumor necrosis factor-α levels increased similarly on days 1 and 5 in severe and non-severe episodes, but on day 30, IL-17a, IL-8, and IL-6 levels were only increased for severe exacerbations. The highest IL-17a level occurred in patients with chronic plus the acute isolation of *Pseudomonas aeruginosa*. At 30 days, severe exacerbations were independently associated with higher levels of IL-17 (Odds ratio (OR) 4.58), IL-6 (OR 4.89), IL-8 (OR 3.08), and hsCRP (OR 6.7), adjusted for age, the bronchiectasis severity index, and treatment duration. Exacerbations in patients with chronic *P. aeruginosa* infection were associated with an increase in IL-17 and IL-6 at 30 days (ORs 7.47 and 3.44, respectively). Severe exacerbations elicit a higher systemic proinflammatory response that is sustained to day 30. Patients with chronic *P. aeruginosa* infection had impaired IL-17a reduction. IL-17a could be a useful target for measuring systemic inflammation.

## 1. Introduction

Bronchiectasis is a chronic inflammatory structural respiratory disease associated with frequent exacerbations of varying severity [1,2]. In general, patients with advanced disease and a high bronchiectasis severity index (BSI) [3] or a high FACED (FEV1, age, chronic colonization, extension, dyspnea) [4] score have an average of two or more exacerbations per year [5], and *Pseudomonas aeruginosa* is the most frequent microorganism involved [6]. It has been clearly demonstrated that exacerbations lead to lung function deterioration [7], poor prognosis [3], and increased mortality [8,9].

Airway and systemic inflammation has been recognized during the stable phase of the disease [10], peaking during exacerbations, although this behavior is not totally parallel in each compartment. In the “vicious vortex” concept, several components beyond microorganisms are hypothesized to affect inflammation and lung damage, with variations in the their interactions explaining the difficulties in exiting the vortex [11]. After an exacerbation, Chalmers et al. [12] reported that there was a reduction in airway but not in systemic inflammation (E-selectin and vascular cell adhesion molecule (VCAM-1)). Other studies have reported a partial reduction in systemic inflammation immediately after antibiotic therapy [13], but few studies have evaluated how much systemic inflammation lasts after an exacerbation, especially when severe. Moreover, there is a lack of information about the role of interleukin (IL)-17. Boyton et al. [14] have implicated the TH17 pathway in bronchiectasis, suggesting that it leads to an immune dysregulation, as reported in cystic fibrosis, which is now considered a TH17 disease. Chen et al. [15] have also reported that there is TH17 activation in the bronchoalveolar lavage fluid of patients with stable bronchiectasis. The importance of systemic inflammation and its persistence is crucial because, beyond its role in disease progression, it could be associated with more adverse events and worse outcomes.

We hypothesized that exacerbations, mainly severe ones requiring hospitalization, are associated with higher systemic inflammation and different speeds of resolution. Proinflammatory cytokines, such as TNF-α, IL-6, IL-8, and IL-1b, were studied, as they have been found elevated in sputum or bronchoalveolar damage in bronchiectasis patients [14]. Moreover, we expected that this would depend on the severity of bronchiectasis, the presence of prior chronic infection, the microorganisms isolated at the time of an exacerbation, and the duration of antibiotic treatment. Therefore, we aimed to evaluate acute systemic levels of proinflammatory cytokines (IL-17a, IL-1β, IL-8, and tumor necrosis factor alpha (TNF-α)) and high-sensitivity C-reactive protein (hsCRP) during bronchiectasis exacerbations and follow-up (days 30 and 60). These were considered with regard to episode severity, acute isolated microorganisms, chronic *Pseudomonas aeruginosa* infection, and BSI score. A steady-state comparison group was also evaluated on day 1, and patients were followed during a 1-year period.

## 2. Study Protocol

### 2.1. Design and Participants

We conducted a prospective observational study at two tertiary care university hospitals of the Spanish National Health Service. Diagnoses of bronchiectasis were based on a clinical history of chronic sputum production, cough, other compatible respiratory symptoms, and the confirmation of bronchiectasis on computed tomography scans of the lungs prior to study enrollment. The following exclusion criteria were applied: severe immunosuppression, such as in solid-organ or bone-marrow transplantation, human immunodeficiency virus infection/acquired immune deficiency syndrome (HIV/AIDS), or receiving chemotherapy or other immunosuppressive drugs (≥20 mg prednisone equivalent per day for ≥2 weeks); active tuberculosis; cystic fibrosis; pulmonary interstitial disease. Patients signed an informed consent form (Biomedical Research Ethics Committee, Hospital La Fe 2011/0342), and were enrolled for 1 year of follow-up. A group of patients with stable bronchiectasis were enrolled that comprised outpatients attending scheduled visits who had no symptoms of exacerbation and had not received oral antibiotics for at least 1–2 months.

### 2.2. Data Collection

We collected data on patient demographic, smoking and comorbidities (e.g., chronic obstructive pulmonary disease, asthma, chronic heart disease). Data related to previous chronic *P. aeruginosa* infection (defined according to Spanish guidelines) [16], number of exacerbations or hospitalization in the previous year, and bronchiectasis severity scores (BSI and FACED) [3,4] were also recorded. Details of usual chronic and concomitant medications used in the last 6 months were collected, including inhaled corticosteroids, inhaled/nebulized antibiotics and macrolides. Antibiotic treatment and its duration in an exacerbation was also recorded.

### 2.3. Microbiological Diagnosis 

We included sputum culture and any other microbiological test performed at the discretion of the attending physician during exacerbations. Sputum samples were considered acceptable if they contained >25 leukocytes and <10 squamous cells per low-power microscope field. The microbiological etiology of exacerbation was defined by any positive result from microbiological investigation, as previously reported [17]. Chronic *P. aeruginosa* infection was defined as three or more consecutive positive cultures from the same microorganisms PPM in a period of at least 6 months in samples separated from each other for at least one month [18].

### 2.4. Cytokine and Biomarker Determination

Blood samples were taken the morning after admission on day 1 and on days 4–5, and 30, and if available, on day 60. The plasma samples were frozen at −80 °C until analysis. We measured hsCRP levels using a microparticle-enhanced turbidimetric assay (CRP Gen.3, Cobas 8000, c701; Roche Diagnostics. Rotkreuz, Switzerland). Plasma cytokine concentrations: interleukin (IL) 1β, IL-6, IL-8, IL-17A, and TNFα were measured with commercial kits (Human Magnetic High-Sensitivity T Cell Mag Ref. HSTCMAG-28SK-04) following the manufacturer’s instructions (Merc. Madrid, Spain) and using Luminex technology (MAGPIX^®^, Berlin, Germany). Data were collected using Luminex xPONENT^®^ software (Berlin, Germany).

### 2.5. Exacerbation Definition and Follow-Up 

Exacerbations were defined as a deterioration in three or more of the following key symptoms for at least 48 h: cough; sputum volume, and/or consistency; sputum purulence; breathlessness and/or exercise tolerance; fatigue and/or malaise; hemoptysis. A clinician also had to determine that a change in bronchiectasis treatment was required [19]. Severe exacerbations were defined as those requiring patients to be admitted to hospital, and non-severe episodes were defined as those treatable on an outpatient basis. The decision to admit was made by the attending physician based on clinical, analytical, and radiological findings. Inpatients were followed up at visits to a specialist clinic on days 30 and 90, and at 1 year after discharge. For outpatients, follow-up was performed on days 7, 30, and 90, and at 1 year. A telephone interview to assess outcomes was used for patients who did not attend follow-up visits.

### 2.6. Statistical Analysis

Statistical analysis was performed using the IBM SPSS 20.0 (IBM Corp., Armonk, NY, USA), and *p*-values ≤ 0.05 were considered statistically significant. Qualitative variables were compared by the χ^2^ test and quantitative variables by ANOVA or the Kruskal–Wallis test. One-way ANOVA or Kruskal–Wallis tests were used for comparisons of more than two groups. Patients with bronchiectasis exacerbations were classified into severe and non-severe groups. Prognostic BSI scores were dichotomized as severe or non-severe (mild and moderate). Blood results are expressed as medians (interquartile range), and for multivariable analyses, were dichotomized by the exacerbation cohort median at day 30 (high levels yes/no). The duration of antibiotic treatment was dichotomized as ≤14 days or >14 days. Finally, several logistic regression analyses were performed using raised levels of IL-17a, IL-8, IL-6, and hsCRP at day 30 as dependent variables. Independent variables were those considered clinically relevant or potential confounders for systemic inflammation, such as age (>65 years: yes/ no), gender, BSI score (severe on non-severe), *P. aeruginosa* isolation (yes/no), prior chronic *P. aeruginosa* infection (yes/no), and exacerbation severity (hospitalization, yes/no). The Hosmer and Lemeshow goodness-of-fit test was used to evaluate the adequacy of the models.

## 3. Results

### 3.1. Patient Characteristics

The cohort consisted of 165 patients followed up for one year. The general characteristics, comorbidities, bronchiectasis etiologies, chronic treatments, severities, and outcomes are shown in Table 1. The control group comprised 34 patients with stable bronchiectasis (no exacerbation for ≥3 months) recruited from our clinic.

### 3.2. Comparison of Patients Treated as Outpatients and Inpatients for Exacerbations

In the exacerbation group, 72 patients were treated as outpatients and 93 were admitted to hospital. Their baseline clinical characteristics are described in Table 2. Of note, more hospitalized patients were older, male, and had chronic comorbid conditions, higher score punctuations obtained in prognostic scales BSI and FACED, but the rates of chronic *P. aeruginosa* infection were similar. The control group was comparable to the outpatient exacerbation group, except for having lower BSI and FACED scores. Patients with severe exacerbations also presented more re-exacerbations at 30 days and had higher mortality at both 30 days and 1 year.

Pathogenic microorganisms isolated in sputum cultures at the time of an exacerbation included *P. aeruginosa* (44; 26.7%), *Haemophilus influenza* (13; 7.9%), Staphylococcus aureus (3; 1.8%), *Moraxella catarrhalis* (3; 1.8%), *Streptococcus pneumoniae* (13; 7.9%), and others (mainly enteric Gram-negative organisms) (24; 14.5%). More than one microorganism grew in the cultures from some patients. No differences existed when comparing isolates between the outpatient and inpatient groups (Table 2). However, more cases of *P. aeruginosa* were isolated in the inpatient group, though without statistical significance. 

### 3.3. Systemic Inflammation in Exacerbations

Blood samples were taken for 90 patients for cytokine and biomarker determination on day 30, but they were only obtained for 75 patients on day 60.

#### 3.3.1. Severe and Non-Severe Exacerbation

**Figure 1:**
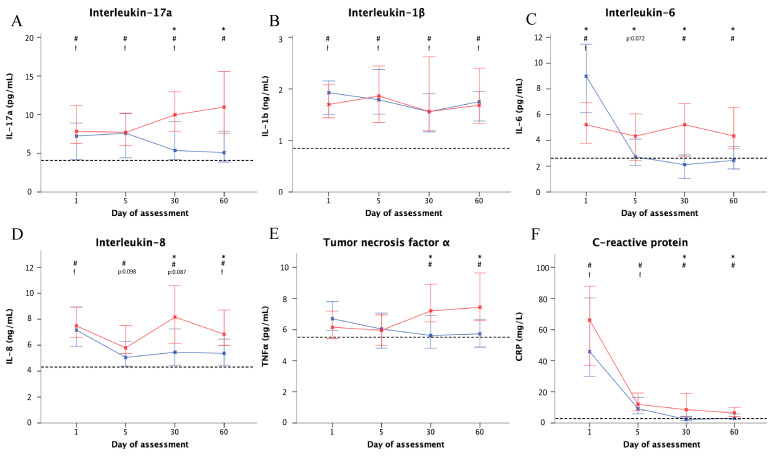
Inflammatory cytokine and hsCRP levels by exacerbation severity and day of assessment. Data are shown for severe exacerbations (inpatients; red) and non-severe exacerbations (outpatients; blue) and by day of assessment (days 1, 5, 30, and 60): (**A**) Interleukin (IL)-17a; (**B**) IL-1β; (**C**) IL-6; (**D**) IL-8; (**E**) TNF-α; (**F**) hsCRP. The dashed line shows the median in control (stable) patients. Blades represent the median and error bars represent 95% Confidence Intervals. * *p* < 0.05 (severe vs. non-severe exacerbations), # *p* < 0.05 (severe exacerbations vs. controls), ł *p* < 0.05 (non-severe exacerbations vs. controls). Abbreviations: hsCRP, high-sensitivity C-reactive protein; TNF-α, Tumor necrosis factor alpha.

Higher levels of inflammation were found during the acute phases of exacerbation compared with the stable patients at baseline and after 30 and 60 days (Figure 1). Levels of the proinflammatory cytokines IL-17a, IL-8, and hsCRP were higher on day 1 compared with the stable patients. However, there were no statistical differences in levels on day 1 between the severe and non-severe exacerbations. Median proinflammatory cytokine levels on day 5 were similar to those on day 1, but there was a decrease in hsCRP levels. On day 30, there was a significantly larger decrease in IL-17a, IL-6, IL-8, TNF-α and hsCRP in outpatients than in patients, and this difference persisted to day 60.

#### 3.3.2. *P. aeruginosa* vs. Other Microorganisms or Unknown Etiology

**Figure 2:**
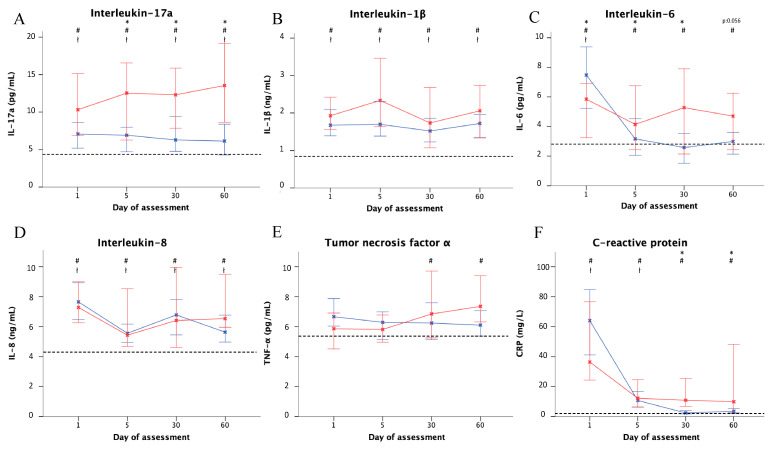
Inflammatory cytokine and hsCRP levels by PA isolation and day of assessment. Data are shown for patients with (red) and without (blue) PA isolation by day of assessment (days 1, 5, 30, and 60): (**A**) IL-17a; (**B**) IL-1β; (**C**) IL-6; (**D**) IL-8; (**E**) TNF-α; (**F**) hsCRP. The dashed line shows the median in control (stable) patients. Blades represent the median and error bars represent 95% Confidence Intervals. * *p* < 0.05 (PA vs. non-PA), # *p* < 0.05 (PA vs. controls), ł *p* <0.05 (non-PA vs. controls). Abbreviations: hsCRP, high-sensitivity C-reactive protein; IL, Interleukin; PA, *Pseudomonas aeruginosa*; TNF-α, Tumor necrosis factor alpha.

Patients with *P. aeruginosa* isolated in their sputum at exacerbation showed a trend for higher IL-17a levels on day 1, reaching statistical significance from day 5 until day 60. Levels were 13.5 (8.63–19.17) with *P. aeruginosa* isolation and 6.13 (3.5–11.7) without *P. aeruginosa* isolation (*p* = 0.03; Figure 2). No statistical differences were found in TNF-α or IL-1β levels between days 1 and 60 regarding *P. aeruginosa* isolation. The levels of hsCRP in exacerbations with *P. aeruginosa* isolation showed significantly higher levels on days 30 and 60 in comparison to cases without *P. aeruginosa* isolation.

#### 3.3.3. Prior Chronic *P. aeruginosa* Infection 

**Figure 3:**
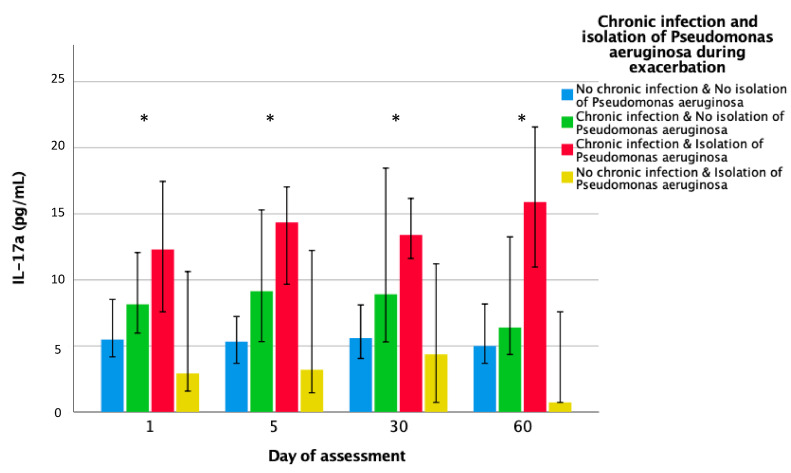
Systemic IL-17a levels by microorganisms isolated at exacerbation. Systemic levels of IL-17a are shown according by chronic PA infection and microorganisms isolated at exacerbation (PA vs. non-PA). * *p* < 0.05 for ANOVA test. No chronic infection and no isolation of *Pseudomonas aeruginosa*, N = 81 (blue); Chronic infection and no isolation of *Pseudomonas aeruginosa*, N = 40, (green); Chronic infection and isolation of *Pseudomonas aeruginosa*, N = 32 (red); No chronic infection and isolation of *Pseudomonas aeruginosa*, N = 12 (yellow). Bars represent the median and error bars represent 95% Confidence Intervals. Abbreviations: IL, Interleukin; PA, *Pseudomonas aeruginosa*.

**Figure 4:**
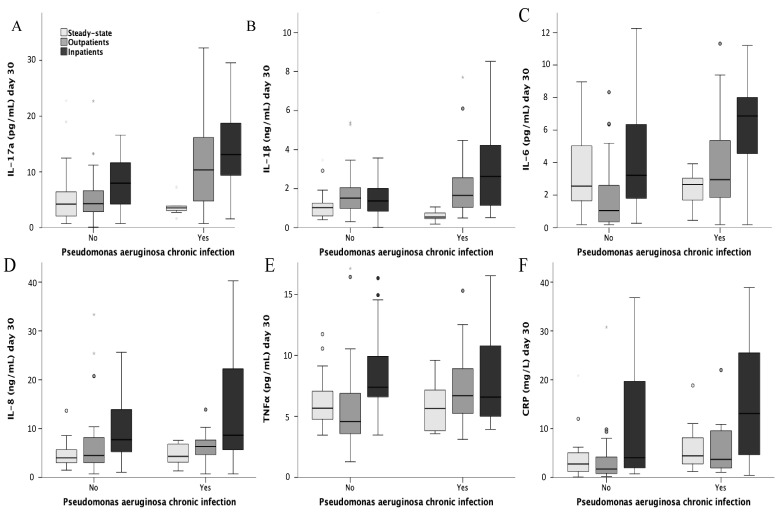
Systemic inflammatory cytokine and hsCRP levels at 30 days by exacerbation severity and chronic PA infection. Boxplot shows the levels of systemic inflammatory cytokines and hsCRP at 30 days in patients with severe and non-severe exacerbations by the presence or absence of chronic PA infection. The box line represents the median, while the boxes include the 25th to 75th percentile. The whiskers span the minimum and maximum values. Points are values that the statistical program automatically considers outliers. (**A**) IL-17a; (**B**) IL-1β; (**C**) IL-6; (**D**) IL-8; (**E**) TNF-α; (**F**) hsCRP. Abbreviations: hsCRP, high-sensitivity C-reactive protein; PA, *Pseudomonas aeruginosa*.

Systemic inflammation during exacerbations was also evaluated according to prior chronic *P. aeruginosa* infection (Figure 3). The highest levels of IL-17a were found in those with both chronic plus acute *P. aeruginosa* isolation. In the box plot figure (Figure 4), systemic inflammation remaining at day 30 is depicted by episode severity and controls. Those with chronic *P. aeruginosa* infection and severe episodes exhibited the highest IL-17a levels.

### 3.4. Multivariable Analysis for Raised Inflammation at Day 30

The cohort median blood values at day 30 used for the multivariable analyses, were as follows: IL-17a, >8.29 pg/mL; IL-1β, >1.56 ng/mL; IL-6, >2.84 pg/mL; IL-8, >6.69 ng/mL; TNF-α, >6.64 pg/mL; hsCRP, >3.67 mg/L. Results were dichotomized as yes/no for high levels. Several regression logistic analyses were then performed using these as dependent variables. Odds ratios (ORs) adjusted for age, gender, BSI, and treatment duration revealed that severe exacerbations were associated with OR-fold increases of 4.58, 4.89, 3.08, and 6.7 for IL-17, IL-6, IL-8, and hsCRP at 30 days. Chronic *P. aeruginosa* infection was associated with a 7.47 OR-fold increase in IL-17a at 30 days and a 3.44 OR-fold increase in IL-6. The Hosmer and Lemeshow goodness-of-fit test was used to evaluate the adequacy of the models [20]. The areas under the receiver–operator characteristic curve for the models are detailed in Table 3.

## 4. Discussion

We showed that there were increases in systemic proinflammatory cytokine and hsCRP levels in patients experiencing exacerbations until day 30, which contrasted with the results for stable patients. The highest inflammatory response was found in severe exacerbations, particularly in patients with both chronic plus acute *P. aeruginosa* isolation during the exacerbation. Proinflammatory cytokine levels increased similarly in both the severe and non-severe exacerbations during the acute phase (days 1 to 5), but IL-17a, IL-6, IL-8, and TNF-α were significantly higher in severe exacerbations by day 30. After adjustments for age, gender, BSI, and treatment duration, the odd ratios for severe exacerbations, being associated with increased proinflammatory marker levels at 30 days ranged from 3.08 to 6.7. The odds for chronic *P. aeruginosa* infection, being associated with increased IL-17 (OR 7.47) and IL-6 (OR 3.44) at 30 days were also high after adjustment.

Compared with the outpatient group, the inpatient group in this study was more likely to be male, older, to have more comorbid conditions, and have higher BSI and FACED scores. The inpatient group also had more prior hospitalizations, but there were no differences in chronic *P. aeruginosa* infection. Overall, the stable patients were comparable to outpatients in all parameters except for having fewer comorbidities and lower BSI and FACED scores. 

Severe and non-severe exacerbations both triggered a higher systemic inflammatory reaction compared with the control group. That pattern mainly involved IL-17a, IL-1β, IL-8, and TNF-α elevations, which is consistent with a response directed to neutrophil chemoattraction and activation. Interestingly, the acute initial elevation in these four proinflammatory cytokines was similar in both the inpatient and outpatient groups, except that hsCRP and IL-6 levels were somewhat higher in the severe inpatient episodes. Moreover, this inflammatory profile was maintained until day 5 of an exacerbation. The pattern associated with IL-17a was also particularly noteworthy, initially showing higher levels in patients with chronic plus acute *P. aeruginosa* isolation at the onset of the exacerbation.

Differences in systemic inflammation appeared from day 5 to days 30 or 60. Instead of showing decreased inflammation after day 5, as occurred in outpatients, the inpatients experienced further increases in IL17a, IL-6, TNF-α, and IL-8 levels. In these severe exacerbations, the activation of inflammation therefore behaved as a gradual process, despite antibiotic treatment, with persistent neutrophil activation and/or an impaired capacity to resolve the inflammation. In fact, levels of inflammation at day 30 remained higher than in the stable patients.

The raised levels of IL-17a, IL-6, IL-8, and hsCRP found in patients with severe exacerbations were adjusted for age, gender, BSI, and duration of antibiotic treatment. Intriguingly, antibiotic therapy for ≥14 days compared to <14 days was not an independent factor associated with systemic inflammation. The optimal duration of antibiotic treatment during an exacerbation is still a matter of debate that is yet to be resolved, but it certainly appears that antibiotics alone are unable to break the cycle of inflammation. This aspect is crucial for treatment strategies and for their potential to affect disease progression. Recently, it has been recognized that bronchiectasis may increase the risk for cardiovascular disease, with systemic inflammation playing a key role [21,22].

The increase in systemic inflammation during an exacerbation has been described in other research, although these have had differences in the monitoring times and cytokines measured. In 48 patients (mean age 43 years) treated for 14 days with a convalescence visit after 7 days of completing treatment without any follow-up beyond one month, Guan et al. [13] observed that CXCL8 and TNF-α levels increased during bronchiectasis exacerbations and partially recovered during convalescence, but that IL-8 levels did not reduce. Courtney et al. [23] also reported that CRP and IL-8 levels fell after an exacerbation (14 days after antibiotic treatment) but increased again 4 weeks later. Chalmers et al. [12] found a decrease in airway inflammation after 14 days of antibiotic therapy, but also showed that this was not associated with a corresponding decrease in serum E-selectin or VCAM-1 levels.

Two particularly important findings emerge from our study: first, there was a sustained increase in systemic inflammation among hospitalized patients after the acute phase; second, the highest elevations and persistence of IL-17a was seen in patients with chronic plus acute isolation of *P. aeruginosa* at exacerbation. Th17 cells have an important role for in the host response against bacteria, and the induction of IL-17 can be beneficial by recruiting inflammatory cells; however, a prolonged induction of IL-17 may lead to persistent inflammation and damage [24]. Boyton et al. [14] observed that an implication of Th17 cell dysregulation in bronchiectasis is that they secrete IL-17 in response to bacterial lung infection. Our findings in severe exacerbations suggest that there is an impairment in downregulating IL-17 and other proinflammatory cytokine levels, such as IL-8 and IL-6, which creates an inappropriate inflammatory milieu [25]. In a murine model, Ritchie et al. [26] demonstrated that IL-17 promoted neutrophil recruitment to the lungs, which may exacerbate lung disease and produce negative outcomes.

Patients with chronic *P. aeruginosa* infection showed the highest IL-17 levels on day 1, and its secretion was further strengthened if *P. aeruginosa* was isolated at exacerbation. In those with acute *P aeruginosa* isolation with no chronic infection, systemic IL-17 levels were not that high. We cannot exclude the real possibility that patients with chronic *P. aeruginosa* infection already had elevated levels of inflammation when their exacerbations started. In fact, IL-17a impairs host tolerance in chronic *Pseudomonas* infection [27]. Munkana et al. suggest that IL-17 has a dual effect in a model of *P. aeruginosa* infection—on the one hand, it recruits neutrophils to the lung for bacterial clearance, but on the other hand, there is a higher production of IL-1β with increases in local neutrophil counts and injury. In patients with stable disease, Chen et al. showed that there was Th17 pathway activation in studies of bronchoalveolar lavage fluid, but they did not find an association with microbiology results or prior *P. aeruginosa* infection. Of note, IL-17a could be a target treatment for modulating immunopathology, as reported in other diseases. Fouka et al. [28] have reported that macrolides are beneficial in controlling Th17 activation.

After hospital admission, there were increases in the percentages of re-exacerbations at 30 days and mortality in the following year. This probably related to the greater and more sustained systemic inflammation [29], which contributes to perpetuating the vortex of infection and inflammation [11]. Even at day 60 after exacerbations, we found that levels of systemic inflammation remained higher than in stable patients.

Our study has several limitations that should be mentioned. First, no bacterial colony counts were quantified in the sputum, but this was because we know that there is a reduction in load after treatment [30]. Second, neither marker of airway inflammation nor elastase were determined in the sputum. However, we were specifically interested in systemic inflammation because this has potential implications for cardiovascular complications. Several comparisons among the groups—severe and non-severe—versus stable patients have been performed, non-corrected, due to the limited number of cases. Finally, the present study was designed to follow a cohort in a “real life scenario,” and as such we only used systemic biomarkers that are more widely available in hospitals. These could have clinical utility for monitoring inflammation during and after exacerbations, with the potential to use IL-17a as a guide for personalized treatments [31].

## 5. Conclusions

Our study has described the pattern of systemic inflammation associated with exacerbations of bronchiectasis. Patients with severe exacerbations who required inpatient care displayed progressive increases in IL-17a, IL-6, and IL-8 that persisted to day 30 after adjusting for patient characteristics, BSI scores, and treatment duration. The highest initial and sustained IL-17a response was found in patients with both chronic plus acute *P. aeruginosa* infection. We conclude that the prolonged systemic activation of IL-17a may lead to persistent local inflammation, epithelial cell alterations, and even distal organ damage. As such, its recognition is key because it can be targeted and treated. Patients with bronchiectasis may benefit from IL-17a monitoring and from novel therapies aimed at clearing or reducing systemic inflammation that could increase the risk of subsequent infection and inflammation.

## Figures and Tables

**Table 1:** Patients characteristics in the whole cohort of exacerbation.

N	N = 165
**Demographics**	
Age	71 (62–78)
Female	103 (62.4)
Smoking	
Current	4 (2.4)
Former	68 (41.2)
Never	93 (56.4)
**Comorbidities**	
COPD	41 (24.8)
Asthma	17 (10.3)
Heart disease	29 (17.6)
**Etiology**	
Post-infective	51 (30.9)
Idiopathic	49 (29.7)
COPD	30 (18.2)
Other	35 (21.2)
**Usual treatments**	
Inhaled steroids	139 (84.2)
Long term macrolides	26 (15.8)
Inhaled antibiotics	35 (21.2)
**Severity**	
FACED	3 (1–4)
Mild	73 (44.2)
Moderate	59 (35.8)
Severe	33 (20)
BSI	10 (7–13)
Mild	18 (10.9)
Moderate	38 (23)
Severe	109 (66.1)
Exacerbation frequency/year	2 (1–2)
**Previous microbiology**	
*Pseudomonas aeruginosa* chronic infection	72 (43.6)
**Microbiology during exacerbation**	
*Pseudomonas aeruginosa*	44 (26.7)
*Haemophilus influenzae*	13 (7.9)
*Streptococcus pneumoniae*	13 (7.9)
Other	30 (18.2)
**Outcomes**	
Exacerbation one month after	21 (12.7)
Exacerbation one year after	90 (54.5)
1-year mortality	14 (8.5)

Data are presented as N (%) or median (interquartile range). Abbreviations: BSI= bronchiectasis severity index; COPD = chronic obstructive pulmonary disease; FACED = F-FEV1, A—Age, C—*Pseudomonas aeruginosa* colonization, E—extension, and D—Dyspnea.

**Table 2:** Patients characteristics.

	Outpatients	Inpatients	*p*	Stable Patients
N	72	93		34
**Demographics**				
Age	65.5 (57–72)	75 (70–80)	<0.001	65.5 (54–76)
Female	55 (76.4)	48 (51.6)	0.001	24 (70.6)
Smoking				
Current	3 (4.2)	1 (1.1)		2 (5.9)
Former	28 (38.9)	40 (43)	0.412	12 (35.3)
Never	41 (56.9)	52 (55.9)		20 (58.8)
**Comorbidities**				
COPD	10 (13.9)	31 (33.3)	0.004	3 (8.8)
Asthma	8 (11.1)	9 (9.7)	0.764	3 (8.8)
Heart disease	5 (6.9)	24 (25.8)	0.002	6 (17.6)
**Etiology**				
Post-infective	19 (30.2)	32 (34.8)		12 (35.3)
Idiopathic	25 (39.7)	24 (26.1)	0.081	18 (52.9)
COPD	7 (11.1)	23 (25)		2 (5.9)
Other	12 (19)	13 (14.1)		2 (5.9)
**Usual treatment**				
Inhaled steroids	60 (83.3)	79 (84.9)	0.778	13 (38.2)
Long-term macrolides	12 (16.7)	14 (15.1)	0.778	3 (8.8)
Inhaled antibiotic	17 (23.6)	18 (19.4)	0.507	4 (11.8)
**Severity**				
FACED	1.5 (1–3)	3 (2–5)	<0.001	2 (1–3)
Mild	48 (66.7)	25 (26.9)		22 (64.7)
Moderate	17 (23.6)	42 (45.2)	<0.001	9 (26.5)
Severe	7 (9.7)	26 (28)		3 (8.8)
BSI	8.5 (5–11)	12 (9–14)	<0.001	5 (2–7)
Mild	14 (19.4)	4 (4.3)		14 (41.2)
Moderate	22 (30.6)	16 (17.2)	<0.001	14 (41.2)
Severe	36 (50)	73 (78.5)		6 (17.6)
Exacerbation frequency/year	1 (0.5–2)	2 (1–3)	0.746	
**Previous microbiology**				
*Pseudomonas aeruginosa* chronic infection	33 (45.8)	39 (41.9)	0.617	12 (35.3)
**Microbiology during exacerbation**				
*Pseudomonas aeruginosa*	14 (19.4)	30 (32.3)	0.065	-
*Haemophilus influenzae*	7 (9.7)	6 (6.5)	0.439	-
*Streptococcus pneumoniae*	4 (5.6)	9 (9.7)	0.330	-
Other	14 (19.4)	16 (17.2)	0.711	-
**Outcomes**				
Exacerbation one month after	4 (5.7)	17 (18.5)	0.017	1 (2.9)
Exacerbation one year after	32 (46.4)	58 (63)	0.035	6 (17.6)
1-year mortality	1 (1.4)	13 (14)	0.004	0 (0)

Data are presented as N (%) or median (interquartile range). Abbreviations: BSI = bronchiectasis severity index; COPD = chronic obstructive pulmonary disease; FACED = F-FEV1, A—Age, C—*Pseudomonas aeruginosa* colonization, E—extension, and D—Dyspnea.

**Table 3:** Multivariable analyses for raised proinflammatory cytokines and hsCRP at day 30.

	IL-17a		IL-1β		IL-6		IL-8		TNF-α		CRP	
Median at Day 30	8.29 pg/mL		1.56 ng/mL		2.84 pg/mL		6.69 ng/mL		6.64 pg/mL		3.67 mg/L	
	*p*	OR (95%CI)	*p*	OR (95%CI)	*p*	OR (95%CI)	*p*	OR (95%CI)	*p*	OR (95%CI)	*p*	OR (95%CI)
**Age (>65 years)**	0.303	1.78 (0.59–5.34)	0.412	1.49 (0.58–3.86)	0.453	0.67 (0.24–1.9)	0.568	0.75 (0.28–2.02)	0.157	1.98 (0.77–5.07)	0.163	0.42 (0.12–1.43)
**Sex (men)**	0.322	0.54 (0.16–1.83)	0.374	0.62 (0.22–1.77)	0.742	0.83 (0.28–2.51)	0.110	2.49 (0.81–7.63)	0.833	1.12 (0.4–3.16)	0.701	0.75 (0.17–3.26)
**BSI (severe)**	0.186	0.42 (0.11–1.53)	0.977	1.02 (0.34–3.01)	0.793	0.86 (0.27–2.73)	0.681	1.27 (0.41–3.88)	0.574	1.36 (0.47–3.99)	0.394	0.53 (0.12–2.28)
**Hospital Admission Last Year**	0.945	1.04 (0.33–3.33)	0.543	0.73 (0.26–2.03)	0.971	1.02 (0.35–3.01)	0.261	0.54 (0.18–1.59)	0.838	1.11 (0.4–3.09)	0.886	0.91 (0.24–3.4)
**Inhaled Steroids**	0.085	0.28 (0.07–1.19)	0.320	1.99 (0.51–7.69)	0.585	1.45 (0.38–5.52)	0.033	5.38 (1.15–25.2)	0.938	1.05 (0.28–3.98)	0.401	0.51 (0.11–2.47)
**Exacerbation with Admission**	0.015	4.58 (1.34–15.71)	0.779	1.15 (0.43–3.13)	0.006	4.89 (1.58–15.14)	0.037	3.08 (1.07–8.86)	0.201	1.89 (0.71–5.04)	0.011	6.7 (1.54–29.23)
**Chronic PA Infection**	0.002	7.47 (2.09–26.76)	0.221	1.93 (0.67–5.51)	0.034	3.44 (1.09–10.84)	0.654	1.29 (0.42–3.94)	0.727	0.83 (0.29–2.37)	0.180	2.61 (0.64–10.6)
**PA Isolation during Exacerbation**	0.444	1.66 (0.45–6.13)	0.535	1.43 (0.46–4.47)	0.991	0.99 (0.30–3.32)	0.429	0.61 (0.18–2.06)	0.689	1.26 (0.4–3.97)	0.108	4.32 (0.73–25.66)
**Treatment Duration >14 Days**	0.257	1.89 (0.63–5.7)	0.726	1.19 (0.44–3.21)	0.495	1.43 (0.51–4.05)	0.436	0.66 (0.24–1.86)	0.977	0.99 (0.37–2.65)	0.209	2.07 (0.67–6.44)

Abbreviations: BSI = bronchiectasis severity index; OR = odds ratio; PA = *Pseudomonas aeruginosa*, CI = confidence interval; IL = interleukin; TNF-α = tumor necrosis factor alpha; hsCRP= high-sensitivity C-reactive protein. The areas under the receiver–operator characteristic curve (AUC) for the models were: IL-17a AUC 0.807 (95% CI 0.72–0.89, *p* < 0.001); IL-1β AUC 0.689 (0.58–80, *p* = 0.002); IL-6 AUC 0.750 (0.65–0.85, *p* < 0.001); IL-8 AUC 0.735 (0.63–0.84, *p* < 0.001); TNF-α AUC 0.67 (0.56–9.78, *p* = 0.005); hsCRP AUC 0.792 (0.68–0.90, *p* < 0.001).
